# Graduate attributes in the disciplines of Medicine, Dentistry and Veterinary Medicine: a survey of expert opinions

**DOI:** 10.1186/1472-6920-9-28

**Published:** 2009-06-05

**Authors:** Anita Laidlaw, Simon Guild, Julie Struthers

**Affiliations:** 1 Bute Medical School, University of St Andrews, St Andrews, Fife, UK

## Abstract

**Background:**

This study was completed as part of a project for the Quality Assurance Agency on the enhancement theme of 'Research teaching linkages: enhancing graduate attributes' in the disciplines of Medicine, Dentistry and Veterinary Medicine. The aims of this investigation were to elucidate a list of desirable research related graduate attributes for the disciplines of Medicine, Dentistry and Veterinary Medicine and provide evidence as to how they could be covered within such curricula.

**Methods:**

Semi structured interviews, symposium breakout sessions and conference workshops were used to define and rank attributes suggested by curricula design experts from the three disciplines. Students graduating from a BSc Medical Science degree program were surveyed to determine how well they felt the curriculum and associated final year project equipped them with the identified attributes.

**Results:**

A list of seven high level attributes which were desirable in graduates wishing to pursue either a professional or research career were identified. 105 students reported that a final year project was particularly effective at developing an understanding of the need to have an inquiring mind and critical appraisal skills whilst other components of their degree course covered team working skills, core knowledge and an understanding of ethics and governance.

**Conclusion:**

This study identified desirable attributes from graduates from medical, dental and veterinary degree programs and provides evidence to support the case for student projects helping to achieve both clinical and research related graduate attributes in medical undergraduates. The project also provides a focus for debate amongst those involved in curriculum design as to whether the attributes identified are those desirable in their graduates and to examine their current curriculum to determine coverage.

## Background

The study reported here was part of a project funded by the Quality Assurance Agency (QAA) Scotland Enhancement theme programme investigating 'Research teaching linkages: enhancing graduate attributes' in the disciplines of Medicine, Dentistry and Veterinary Medicine [1]. One component of the project investigated research related graduate attributes and aimed to determine and define what attributes were desirable in graduates from these three professional degrees.

There has been debate regarding the shortfall in graduates from professional degrees who opt to include research in their career plans. The Selborne Report raised concerns over the future of Veterinary Medicine research [2], whilst the new draft version of the General Medical Councils (GMC) Tomorrow's Doctors guidelines includes the high level outcome of 'The doctor as a scholar and scientist' under which fall research skills such as 'Formulate simple relevant research questions in biomedical science, psychosocial science or population science and design appropriate studies or experiments to address the questions' [3]. Therefore there are concerns over the potential of future professional graduates to carry out research relating to their careers. Part of this concern focuses on the lack of experience and training during the undergraduate years. The difficulty lies in ensuring that appropriate training is given during degree programs to equip interested students with the skills required whilst not specifically training all undergraduates for this career. The 'product' of a vocational degree course such as Medicine, Dentistry or Veterinary Medicine should be a competent professional equipped for their first day in practice, therefore research skills may not be necessary. So should these crowded curricula be pressured further to 'fit in' research as well as professional attributes? Would ensuring that research related graduate attributes are covered exert further pressure on curricula?

Professional attributes should already be covered within the curriculum as required by the governing bodies the General Medical Council (GMC) [4], the General Dental Council (GDC) [5] and the Royal College of Veterinary Surgeons (RCVS) [6] but what research attributes are desirable of such graduates? Identification of these attributes would enable curriculum designers to map them onto current teaching and recognise gaps in coverage.

Also of interest is the opinion of students graduating from such professional/vocational programs as to whether the courses do cover the attributes discovered. The BSc in Medical Sciences at St Andrews is a three year Honours degree program, after which graduates move on to their clinical years at other Medical Schools. The program itself is designed to provide 'an excellent scientific foundation for clinical practice' [7] and therefore should cover both professional and research practice. But would students completing such a course agree? A major component of the course, similar to other Honours degree programs, is the completion of a final year project. We investigated whether the degree program, and the final year project in particular, helped to equip graduates from the BSC Medical Sciences with the attributes that experts in the field thought were vital for a successful professional and research career.

The first aim of this study was to identify what research related graduate attributes were desirable in graduates from Medical, Dental or Veterinary degrees. A secondary aim was to determine whether such research related attributes can be covered in a curriculum whose content is governed by regulating bodies. The third and final aim was to investigate student opinion as to whether a course designed to provide a scientific foundation in Medicine would equip graduates with the identified attributes.

## Methods

### Defining attributes

Semi structured interviews were carried out with key staff members responsible for curriculum design from all Medicine, Dentistry and Veterinary Medicine schools in Scotland. Interviewees were purposively selected as being involved with curriculum design within their school. Further interviews were carried out with selected staff from English schools. These schools (from all three disciplines) were selected to provide a wide range of views from different teaching and learning environments. The interview was structured around the Quality Assurance Agency enhancement theme 'Research-Teaching Linkages: enhancing graduate attributes' aims, and was piloted with two participants from different Medical schools, after which minor revisions were made.

A total of 24 interviews were completed, either in person or over the telephone. Sampling was concluded once all Scottish schools and the selected English school representatives were interviewed. Interviews were recorded and transcribed. Of interest to this study was the question: 'Can you name 10 key research attributes that graduates from your course should have?'

Qualitative analysis of the transcripts was carried out using a grounded theory approach [8]. Two of the authors (AL and JS) examined the suggestions put forward by all interviewees and clustered them around emerging themes. This work was carried out in NVivo software. The themes were refined by both authors into a list of attributes which described the suggestions clustered under them. For example, the following quotes from a Dental and Veterinary interviewee were both refined, along with others to the attribute 'Inquiring mind/curiosity':

Quote 1: 'Asking questions, as in a method of learning an enquiring mind'

Quote 2: 'Encouraging aspects of the personal and professional behaviour such as being interested in a problem enough to try and find out more about it'

Delegates (n = 58) at the symposium 'Research Teaching Linkages in the disciplines of Medicine, Dentistry and Veterinary Medicine', (Edinburgh, January 2008) discussed the list of attributes during small group breakout sessions. Group composition was balanced to ensure they contained representatives from each discipline. Using a variation of nominal group technique, participants were asked to discuss and define the predefined list of attributes and rank them in order of preference from a professional standpoint and then again from a research standpoint. Groups were invited to discard any attributes they thought were not vital. During collation of the results from all groups, attributes ranked by less than three out of the five groups at the symposium were discarded. Attributes that were deemed vital by three or more groups at the symposium had their average ranks calculated. This provided a final list of attributes, the definitions of which were provided by the groups. The definitions were further refined at a QAA Enhancement Themes conference (Heriot Watt University, March 2008) workshop aimed at the three disciplines.

### Student survey

Final year students (n = 104) of the BSc Medical Sciences degree program at the University of St Andrews were asked to complete a questionnaire to evaluate the course as part of the curriculum review process. The part of the questionnaire relevant to this study focussed on how well equipped they felt with the graduate attributes defined previously in the study. For example questions asked whether completion of either the degree program or the final year project module had given them 'an understanding of the evidence base for practice', or 'has made you aware of the need for an inquiring mind' (1 = agree - 5 = disagree).

The data was investigated for normality and statistical analysis was carried out using appropriate tests in SPSS v 14.

Ethical approval was granted by the Bute Medical School Ethics Committee.

## Results

### Graduate attributes – ranks and definitions

Interviews with 24 staff members from Medical, Dental and Veterinary schools yielded 122 graduate attributes, an average of five per interviewee. There were no differences between the three disciplines in terms of the attributes suggested by faculty members. Qualitative analysis (described above) of the interview transcripts resulted in 16 higher level attributes.

Discussion at the Research Teaching Linkages symposium breakout sessions resulted in nine attributes being thought to be vital for a professional career and 10 for a research career. Table 1 presents the final lists of attributes together with the average rank given to that attribute by the symposium breakout groups.

**Table 1:** Average rankings for attributes from professional and research standpoints

**Professional standpoint**		**Research standpoint**	
**Attribute**	**Average rank (± standard error)**	**Attribute**	**Average rank (± standard error)**
Core knowledge	1.33 ± 0.33	Inquiring mind/curiosity	1.8 ± 0.58
Critical appraisal	3.67 ± 1.76	Core knowledge	3.25 ± 1.60
Understanding of the evidence base for practice	3.67 ± 1.76	Critical appraisal	3.4 ± 0.93
Inquiring mind/curiosity	4.67 ± 1.86	Understanding of the evidence base for practice	4.0 ± 2.12
Ability to communicate	5.0 ± 3.51	Ability to work in a team	4.33 ± 1.76
Ability to work in a team	5.33 ± 2.60	Understanding of ethics and governance	4.4 ± 1.44
Understanding of ethics and governance	5.67 ± 0.88	Ability to communicate	5.25 ± 2.72
Appreciate the need for continuing professional development	7.67 ± 3.76	A broad understanding of research methods	5.8 ± 1.53
IT skills	9 ± 1.15	Appreciate the need for continuing professional development	6.0 ± 3.11
		Ability to generate research data	7.0 ± 3.06

Average ranking for graduate attributes selected by at least three of the five symposium breakout groups for inclusion into their lists from a professional career or a research career standpoint (mean ± standard error).

As can be seen from Table 1, the top seven attributes in both lists were the same, although they were not ranked in the same order. The average combined ranking of these top seven attributes and their final definitions (further refined by the QAA Enhancement Themes conference workshop attendees) are shown in Table 2.

**Table 2:** The final top seven attributes common to both professional and research careers and definitions.

**Attribute**	**Average rank (± standard error)**	**Definition**
Inquiring mind/curiosity	2.25 ± 0.65	A motivation to understand and explain. It should be fostered and encouraged during the undergraduate years by giving the tools to do this, such as developing a hypothesis and testing it.
Core knowledge	2.43 ± 0.95	Core knowledge is continually developing and there should be an understanding of this in graduates. A function of core knowledge is that it enables individuals to ask questions and challenge facts.
Critical appraisal	3.50 ± 0.80	This is the ability to understand evidence, by which we mean analyse, criticise and synthesise it.
Understanding of the evidence base for professional practice	3.86 ± 1.32	In all practitioners an awareness of the need for evidence supporting their practice is vital.
Understanding of ethics and governance	4.25 ± 0.94	This includes an understanding of ethical and legislative issues of professional and research practice such as ethical committees and Home Office powers.
Ability to work in a team	4.83 ± 1.42	The team is loosely defined and could include multi-professional teams in either a research or clinical setting. This should also include an appreciation of different team roles and their importance in ensuring teams work effectively.
Ability to communicate	5.14 ± 1.97	This broad attribute covers all aspects of communication. It incorporates a variety of individuals such as patients, co-workers, experts in the field or the general public. It also encompasses communication using various media, one to one interactions, public speaking, written notes, leaflets or journal articles. The theoretical as well as the practical elements of communication should be covered.

Combined rankings of the top seven graduate attributes shared between the professional and research careers and definitions (mean ± standard error).

### Survey results

Response rate of the student questionnaire was 100%. Table 3 shows average student ratings of how well they felt their degree program and specifically the final year project had equipped them with the seven graduate attributes.

**Table 3:** Average student scores for how well the degree program or final year project equipped them with the seven attributes identified by this study.

**Attribute**	**Degree Average (± standard error)**	**Project Average (± standard error)**	**t**	**df**	**Sig (2-tailed)**
Inquiring mind/curiosity	2.94 ± 0.081	3.35 ± 0.073	3.75	205	0.001
Core knowledge	3.28 ± 0.072	2.88 ± 0.10	-3.20	206	0.002
Critical appraisal	2.74 ± 0.077	3.16 ± 0.082	3.73	206	0.001
Understanding of the evidence base for professional practice	2.81 ± 0.82	2.98 ± 0.97	1.26	206	0.209
Understanding of ethics and governance	2.51 ± 0.13	3.01 ± 0.85	-3.20	206	0.001
Ability to work in a team	3.13 ± 0.092	1.62 ± 0.14	-9.17	207	0.001
Ability to communicate	3.13 ± 0.09	3.12 ± 0.09	0.02	208	0.985

Student scores for whether the degree program or final year project equipped them with the seven attributes (mean ± standard error). Independent samples t-tests show whether the degree program or final year project scored higher for any particular attribute.

Degree program average scores for all attributes were significantly higher than 2, which would be a 'neutral' response (one sample t-test, results not shown). The final year project was also thought to equip students well with all attributes, apart from team working where the average score was lower than neutral (t = 2.77, df = 102, P = 0.007).

It can be seen from Table 3 that there were differences between the ratings for the degree program and the final year project. Specifically the research project was thought to be better at training the students to understand the importance of having an inquiring mind and for improving critical appraisal skills compared to the degree program. The degree program itself was thought to be more effective at equipping students with core knowledge, team working and an understanding of ethics and governance. The degree program and the final year project were thought to be equally good at ensuring graduates understood the evidence base for practice and improving their ability to communicate.

## Discussion

Interviews with faculty members with responsibility for curriculum design from all three disciplines and ranking by further experts in the area resulted in two lists of attributes, one desirable in a graduate pursuing a professional career, the second in a graduate pursuing a research career. The definitions agreed in the study (Table 2) provide a useful focus for debate amongst curriculum designers as to whether the opportunity to develop such attributes, are available within their program. The definitions may also help the developers of teaching sessions to design elements to foster and encourage these attributes amongst their undergraduates.

Interestingly the top seven attributes in both lists were the same, although not ranked in exactly the same order of preference. This suggests that such graduate attributes are high level skills which would be applicable to both research and professional situations. This finding has implications for curricula design in Medical, Dental and Veterinary degree programs. Curricula in all three disciplines are prescribed by guidelines issued by the governing bodies, the GMC [4], GDC [5] and RCVS [6] which are focussed towards the production of graduates competent to pursue a professional career, although they do suggest graduates be aware of the evidence base for practice and have an understanding of research methods. This study suggests that the two career aims (professional and research) are not mutually exclusive in terms of curriculum design and opportunities for learning within the curriculum. Indeed it may be the context in which the attributes are taught/encouraged which results in the student perceiving the session as being professional or research related [1]. This means that designing courses which cover research related skills can be accomplished without adding further pressure to already crowded curricula. This does raise further questions as to how graduates can be encouraged to consider incorporating research into their careers, especially within veterinary research where the Selbourne report noted there was a lack of such people [2].

A BSc program in Medical Sciences may not have precisely the same aims as the standard five year MBChB and this is one limitation of this study, as graduates may have a slightly different focus in their careers. The course at the University of St Andrews provides the basis for entrance to the third year of an MBChB course, but also attempts to deliver an understanding of the scientific basis of Medicine and therefore should be equipping its graduates with the research related attributes determined in the previous part of the study. The results show that the final year students considered that the degree program as a whole, did provide them with the opportunity to develop all of the top seven attributes identified in this project as being common to both professional and research careers. The final year project component of the course was viewed as being particularly effective at equipping students with the ability to understand the importance of having an inquiring mind and developing their critical appraisal skills. Similar outcomes for research projects were shown in a qualitative study in science undergraduates and their faculty advisors by Seymour et al [9]. They noted that the research project increased application of knowledge and skills such as critical thinking, changed attitudes, behaviours (such as increasing curiosity and initiative) and personal/professional gains (such as increasing confidence in communicating their work to others) [9,10]. Interestingly the final year project in the St Andrews curriculum was not thought to improve team working skills within the students as much as other attributes. This may be due to the library based nature of the project undertaken by many of our students, rather than being laboratory based, where they would be working alongside others such as post-doctoral researchers or PhD students. Overall though this project provides further evidence as to the benefits of undergraduates taking part in research projects, but extends the previous work in pure science degrees to a Medical Sciences degree which includes a vocational element. The research project therefore is an excellent example of a method that can link research and teaching in the minds of the undergraduate, a vital component of the research-teaching nexus [1]. As the new draft version of the GMCs Tomorrow's Doctors guidelines includes the high level outcome of 'The doctor as a scholar and scientist' [3], this may be one way to cover such areas within the medical curriculum.

Other limitations of this study include the sample of interviewees, which included individuals from all Scottish Medical, Dental and Veterinary schools and a selected sample from English schools. The English schools were selected for participation on the basis of achieving representatives from a wide range of curriculum types although it is not an exhaustive sample. We acknowledge that this may have impacted on the views that were obtained.

Data analysis employed both qualitative and quantitative methods. The development of the list of refined attributes from the interview suggestions utilised NVivo software which may have affected coding and clustering of data and therefore the emerging themes. The process was however carried out by two authors (AL and JS) who achieved consensus.

This study is timely as the Higher education sector becomes ever more globalised. Many Veterinary schools in the UK comply with European Association of Establishments for Veterinary Education curriculum guidelines [11]. The Tuning project [12] is also ongoing and has the expansive aim of tuning educational structures and courses in higher education across Europe. This includes agreeing generic graduate competencies which should be outcomes for all degree programs. The Thematic Network on Medical Education in Europe (MEDINE) is working within the Tuning project among others [13]. One piece of research carried out by the MEDINE group was an online survey investigating how vital medical educators viewed the generic Tuning competencies. Interestingly, of the 29 generic Tuning competencies, the 15 which were ranked most vital by medical educators across Europe were similar to the top seven attributes identified in this study [14]. The seven attributes defined in the current project are possibly higher level and therefore incorporate one or more of the Tuning project generic competencies. One of the Tuning project generic competencies is 'research skills': this study would argue that in fact all of the seven attributes identified in this project are, in fact 'research skills'. The results of this study add to the current discussions around identifying and agreeing a common range of graduate attributes in the wider context of European education in the disciplines of Medicine, Dentistry and Veterinary Medicine.

## Conclusion

Curriculum designers in Medicine, Dentistry and Veterinary Medicine can utilise the seven graduate attributes identified in this study as a means of ensuring that they produce graduates who are equally well equipped for either a professional or research career. By mapping these core attributes to current learning activities they can ensure that there are opportunities within their curriculum for students to develop and practice these skills. The definitions of these attributes will allow a focus for discussion on the opportunities available within the different curricula.

Results of the student study confirmed the belief that involving students in a research project was an excellent way to equip them with many of the identified attributes. Students completing a BSc Medical Sciences reported that the final year project was particularly effective in developing an inquiring mind and the skills of critical appraisal.

## Competing interests

The authors declare that they have no competing interests.

## Authors' contributions

AL was a member of the original project team, carried out interviews, facilitated symposium breakout sessions and drafted this article. SG derived the initial QAA project proposal and recently presented some of the results of the project at the AMEE conference, Prague. JS was project director of the original QAA project, carried out interviews, facilitated symposium breakout sessions and commented on drafts of this article. All authors read and approved the manuscript.

## Pre-publication history

The pre-publication history for this paper can be accessed here:


